# Improved therapeutics of modified mesenchymal stem cells: an update

**DOI:** 10.1186/s12967-020-02234-x

**Published:** 2020-01-30

**Authors:** Dickson Kofi Wiredu Ocansey, Bing Pei, Yongmin Yan, Hui Qian, Xu Zhang, Wenrong Xu, Fei Mao

**Affiliations:** 1 Key Laboratory of Medical Science and Laboratory Medicine of Jiangsu Province, School of Medicine, Jiangsu University, 301 Xuefu Road, Zhenjiang, 212013 Jiangsu People’s Republic of China; 2 Department of Clinical Laboratory, Suqian First Hospital, Suqian, 223800 Jiangsu China; 3 Directorate of University Health Services, University of Cape Coast, Cape Coast, Ghana

**Keywords:** Mesenchymal stem cells, Modification, Genetic, Preconditioning, Therapy

## Abstract

**Background:**

Mesenchymal stromal cells (MSCs) have attracted intense interest due to their powerful intrinsic properties of self-regeneration, immunomodulation and multi-potency, as well as being readily available and easy to isolate and culture. Notwithstanding, MSC based therapy suffers reduced efficacy due to several challenges which include unfavorable microenvironmental factors in vitro and in vivo.

**Body:**

In the quest to circumvent these challenges, several modification techniques have been applied to the naïve MSC to improve its inherent therapeutic properties. These modification approaches can be broadly divided into two groups to include genetic modification and preconditioning modification (using drugs, growth factors and other molecules). This field has witnessed great progress and continues to gather interest and novelty. We review these innovative approaches in not only maintaining, but also enhancing the inherent biological activities and therapeutics of MSCs with respect to migration, homing to target site, adhesion, survival and reduced premature senescence. We discuss the application of the improved modified MSC in some selected human diseases. Possible ways of yet better enhancing the therapeutic outcome and overcoming challenges of MSC modification in the future are also elaborated.

**Conclusion:**

The importance of prosurvival and promigratory abilities of MSCs in their therapeutic applications can never be overemphasized. These abilities are maintained and even further enhanced via MSC modifications against the inhospitable microenvironment during culture and transplantation. This is a turning point in MSC-based therapy with promising preclinical studies and higher future prospect.

## Background

The introduction of cell therapy has made dramatic contribution to science and continue to expand in value and utility in regenerative medicine and disease therapeutics. Experimental and clinical applications of cell therapy has covered many diseases including cardiovascular conditions [1–3], Parkinson’s disease [4, 5], osteoarthritis [6], diabetes [7, 8], neurological conditions [9], wounds [10], malignancies [11], among others. The special characteristic properties of MSCs have been exploited in countless research field with fascinating outcomes. The interest in these cells grows exponentially due to their flexibility of being used alone, with other substances, as a carrier, and in combined therapy. MSCs have also been modified to exert specific effects and/or induce enhanced functionalities of itself or other substances. Modified MSCs have been applied across several conditions including malignancies, with the assertion that, pretreated MSCs demonstrate increased differentiation efficacy, improved paracrine functions, superior cell survival, and increased ability to home at site of injury [12–17]. In this review, we bring an up-to-date report on genetic modification and preconditioning of MSCs as applied across several human diseases. We discuss the progress, challenges, and future perspective of this promising field.

## Mesenchymal stem cells

Generally, MSCs are plastic adherent population of cells, having self-renewing ability and capable of differentiating into adipogenic, osteogenic and chondrogenic lineages, among others. They possess intense immunomodulatory property but low immunogenicity. All MSCs isolated from various sources have common characteristic functions of inducing regeneration as well as maintaining general tissue homeostasis due to their special inherent properties including the ability to home at target sites [18]. To date, the clinical and experimental utility of MSCs span across countless number of diseases and conditions, accompanied with promising outcome. MSC based therapy has attracted a lot of interest and continue to expand in its application. Notwithstanding, transplanted MCSs are mostly unable to reach their full therapeutic potential partly due to their inability to sufficiently migrate to the target site, encounter hostility within the transplanted microenvironment causing reduced engraftment time, and lack differentiation and proliferative ability due to lengthy culture period [19–21]. These challenges among others form the focus of MSC modification.

**Fig. 1:**
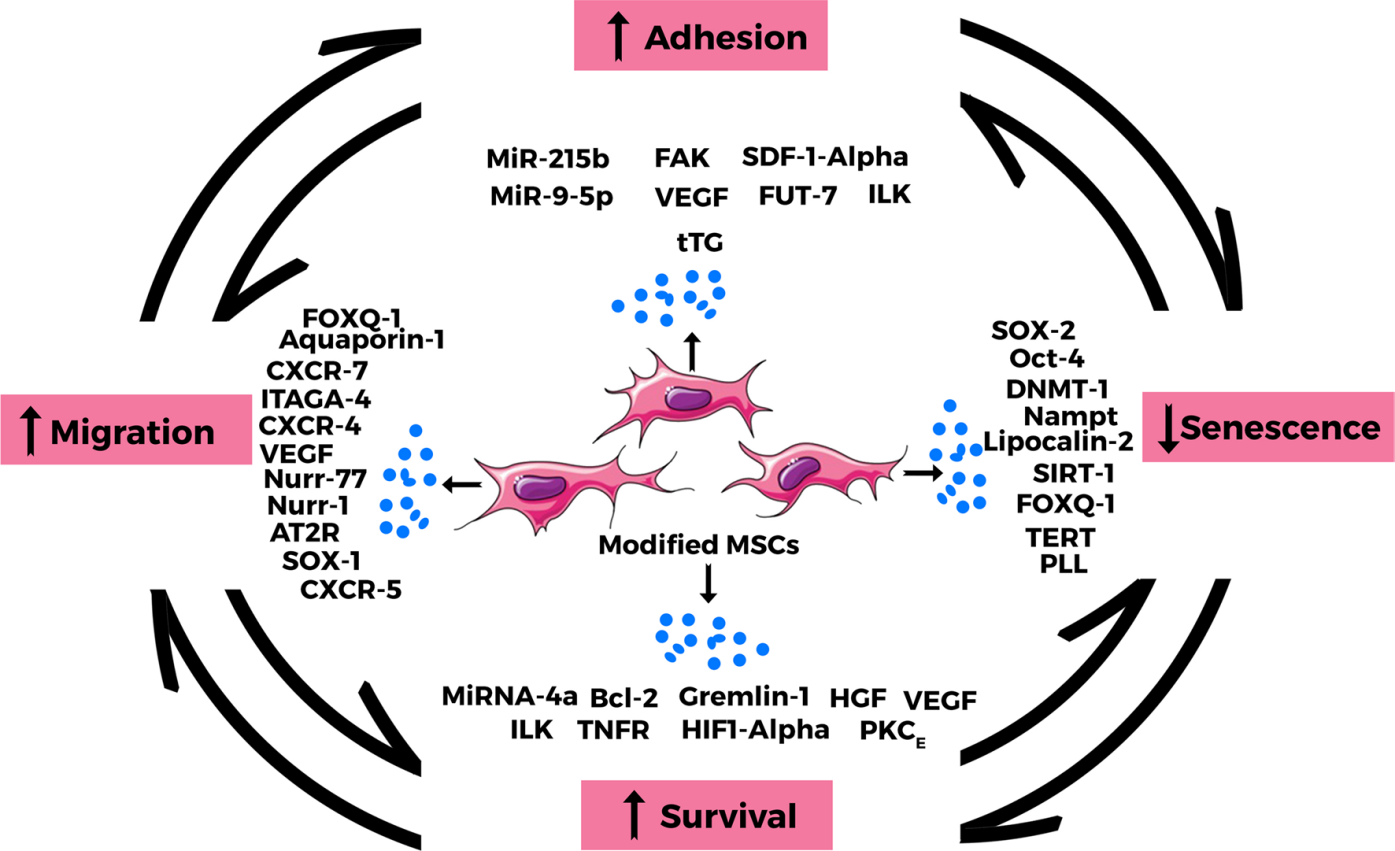
The focal points of enhancement of MSC properties during modification. MSC modification is geared towards improving their inherent therapeutic properties via enhanced migration, adhesion, survival and reduced senescence. These properties are interdependent and greatly influenced by pretreatment factors and expressed cytokines

Regardless of the immense contributions and successes associated with MSC-based therapy, there has always been the need for improvement to enhance the inherent properties and circumvent the confronted challenges. This quest has made room for the modification of MSCs, resulting in an improved and highly specific therapeutic effects in many experimental studies while clinical trials are still in their early stages with preliminary aims to evaluate safety, efficacy and feasibility [22–24]. The application of both genetic and preconditioning modifications involve three basic steps, MSC isolation and propagation, followed by preconditioning or transfection with specific gene or molecule, and finally the introduction of the modified MSCs into test subject. Subsequent discussion in the text will focus on genetic and preconditioning modification approaches that modulate survival, migration, adhesion and senescence of MSCs. These four focal improvement of MSC properties towards better therapeutics effects is shown in Fig. 1.

## Genetic modification

In the genetic modification of MSCs, a constructed gene cassette is loaded into a vector for easy entry into the MSC. Once inside the MSC, it produces or overexpresses certain specific genes. The transgene expression could either remain constant resulting in characteristic synthesis of specific molecular proteins or could be regulated via a gene switch [25].

### Improving migration

Genetic modification of MSCs seeks to improve cellular survival, increase migration, homing and adhesion to target sites, as well as avert poor MSC division and growth (senescence). On the basis of enhancing MSC migration, the induction of CXC chemokine receptors 1, 4 and 7 overexpression has been employed [26]. While CXCR4 and CXCR7 serves as specific receptors for one of the most powerful chemokines concerned with cellular migratory processes, which is the stromal cell-derived factor 1 (SDF-1) [27–29], CXCR1 is rather a receptor for interleukin-8 (IL-8) [30]. These studies report that the overexpression of CXCR4/CXCR7 in the adipose tissue-derived MSCs, promotes their paracrine, proliferative and migratory abilities. CXCR7 is required not only for the migration and proliferation of MSCs but also for their angiogenesis [27]. This modification wields powerful therapeutic impact and has been applied in liver and kidney studies [31, 32], cerebral ischemia reperfusion models [33], infarcted myocardium [21], among others. Du and colleagues document that, CXCR4 overexpression enhances the mobilization and engraftment of MSCs into rat liver grafts, during which the MSCs encourage the early regeneration of the remnant liver not by direct differentiation but probably via a paracrine mechanism [31]. In a rat cerebral ischemia/reperfusion model, it is reported that both CXCR-4 and CXCR-7 receptors were co-expressed in bone marrow-derived MSCs and synergistically promoted their migration even though the effect of CXCR-7 was stronger than that of CXCR-4. The migrated MSCs promoted autocrine and paracrine signaling of SDF-1α [33]. Other over-expression modifications that adequately improve MSC migration and homing include the nuclear receptors Nurr1 and Nur77 [34, 35], aquaporin-1 gene [36] and integrin subunit alpha 4 (ITGA-4) [37]. In their study to identify genes involved in MSCs migration, Maijenburg and colleagues record that the nuclear receptors Nur77 and Nurr1 show the highest expression in migratory MSC. Further analysis of the cell cycle shows a reduction in the proportion of cells in S-phase (in Nur77 and Nurr1 expressed cells) as compared with control cells [35]. The promoted migratory ability of MSCs as demonstrated by the overexpressed Aquaporin-1 and CXCR-4, is partly via the activation of the Akt and Erk intracellular signaling pathways [36].

### Improving adhesion

Optimum adhesion of MSCs is crucial in the determination of their proliferation and viability on surfaces of substrates and contribute to cellular engraftment and tissue regeneration. This adhesion is known to be linked with integrins, which control cell-extracellular matrix (ECM) and cell–cell adhesion mechanisms through adhesion molecules and ECM binding [38]. In this way, MSC adhesion ability, alongside other inherent properties are improved by the expression of integrins and focal adhesion complex. For example, when MSCs were genetically modified to overexpress integrin-linked kinase (ILK), their survival rate increased by 1.5-fold and the phosphorylation of ERK1/2 and Akt in the transfected MSCs increased by approximately three and twofold respectively. The adhesion rate also increased by 32.2% when transplanted into an ischemic myocardium model, with a higher retention rate of approximately fourfold compared to the unmodified MSCs. The enhanced cell survival and adhesion led to improved myocardial damage recovery [39]. Again, surface modifications with biomimetic extracellular matrices [40], poly(dimethylsiloxane) treated with glutaraldehyde and (3-aminopropyl)triethoxy silane [41], and certain bio-active molecules [42], also resulted in a stronger MSC adhesion and proliferation. This technology is of great significance in application across many conditions including cardiac diseases where it is reported to improved micro-vessel density, left ventricular function, and reduce infarct size [43]. Li and colleagues also report that, the overexpression of miR-9-5p by MSCs did not only cause increased migration but also improved focal adhesion [44]. To further elaborate the mechanism underlying this effect, the study screened the target genes of miR-9-5p and report that, CK1α and GSK3β (inhibitors of β-catenin signaling pathway), were direct targets of miR-9-5p in MSCs hence the overexpression of miR-9-5p upregulates β-catenin signaling pathway. In another study, the enhanced adhesion dynamics were confirm to be regulated by focal adhesion kinase (FAK) and Rac1 in a vascular endothelial growth factor (VEGF) induced MSCs migration experiment on neural differentiation [45]. Additionally, glycol-engineering of MSCs overexpressing α(1,3)fucosyltransferase, was confirmed to be safe, producing efficient MSC homing and adhesion in an ischemia reperfusion model. Whereas coupling using 19Fc[FUT7(+)] enhanced cell capture on recombinant P-selectin, α(1,3)fucosylation was necessary for robust binding to E-selectin and inflamed endothelial cells under shear; together enhanced the stem cell engraftment [46].

### Improving survival

MSCs are also modified to increase their survival time against the unfavorable microenvironment within which they are administered or cultured. This makes room for the cells to thrive longer enough to illicit sufficient therapeutic effects. Integrin-linked kinase (ILK) overexpression in MSCs increased their survival and angiogenesis via AKT and mTOR signaling pathways in an infarcted myocardium. It was noticed that the ILK-overexpressed MSCs increased MSCs survival at day 4, and angiogenesis at week 3 post transplantation [47]. A similar outcome was recently demonstrated by Mao and colleagues who report an increased self-renewal and survival of MSCs due to over-expression of ILK under hypoxic condition. The ILK triggers IL-6 secretion and consequently JAK2/STAT3 and Wnt signaling pathways activation in the process [48]. Hypoxia inducible factor 1α (HIF1α) transfected MSCs are also known to enhance MSC viability and survival under hypoxia [49], protects MSCs against oxygen–glucose deprivation-induced injury [50], and even help in MSCs mobilization into peripheral blood [51] resulting in an overall improved injury repair [52, 53]. Lv and colleagues transfect rat MSCs with small interfering RNA Hif1a gene under hypoxia, after which cell viability, apoptosis and expression of HIF1A were analyzed. The hypoxic condition elevates the viability of MSCs and up-regulates HIF1A expression, which consequently promotes survival and suppresses apoptosis even under normoxia. This study further asserts that, the possible underlying mechanisms of this effect may involve the HIF1A-suppressed p53 pathway [49].

Hepatocyte growth factor (HGF) is another significant cytokine that participate in angiogenesis, anti-inflammation and anti-apoptosis. MSCs overexpressing HGF are reported to have high therapeutic influence in cardiovascular diseases [54], liver injuries [55], lungs conditions [56], stabilizing endothelial barrier function [57, 58], skeletal muscle tissue regeneration [59], and even regeneration of damaged neurons in a Parkinson’s disease model [60]. Jang and colleagues investigate the protective role of HGF gene-transfected MSCs in acetaminophen induced hepatocytes injury. They report that, the HGF gene-transfected MSCs increase cell survival and expression of anti-apoptosis protein Mcl-1, resulting in enhanced hepatocyte proliferation and protection [55]. In another investigation aimed at evaluating the cardio-protective effects of MSCs overexpressing HGF in a mouse model of myocardial infarction, umbilical derived-MSCs treated with HGF-conditioned medium were harvested and transplanted. Initial analysis indicated that, MSCs overexpressing HGF show less cell apoptosis in response to hypoxic challenge, and express higher levels of other cytokines like VEGF, EGF and bFGF. Upon post transplantation analysis, these MSCs were associated with greatly improved heart function characterized by less cardiomyocyte apoptosis, improved cardiomyocytes proliferation and enhanced angiogenesis [54]. Other MSCs modifications known to enhance their survival include but not limited to overexpression of Gremlin1 [61] and protein kinase Cɛ [62], co-overexpression of Bcl-2 and VEGF [63], upregulation of TrkB [64], inhibition of mircoRNA-34a [65], Cripto stimulation [66], and tumor necrosis factor receptor (TNFR) gene transfection [67]. For instance, Bao and colleagues investigated the enhancement of MSCs survival by transfecting the cells with TNFR gene, causing overexpression of TNFR and the binding of TNF-alpha. Two weeks post-transplantation analysis indicates an enhanced engrafted MSC survival in the infarcted myocardium alongside other indicators of improved left ventricular function [67].

### Reducing premature senescence

Another critical focus of genetic modification of MSCs is to prevent premature senescence of cultured or transplanted cells. Cellular senescence, which essentially refers to the irreversible cellular proliferation arrest, greatly contributes to reduced MSC functions. Genetic modification of MSCs involving Sox2 and Oct4 genes overexpression, have been shown to efficiently improve differentiation and proliferation potential of transplanted MSCs [68], as well as their anti-inflammatory effects [69]. To enhance MSCs stemness and proliferation, Han and colleagues introduced human Oct4 and Sox2 into the cells to confer higher expansion and differentiation abilities via liposomal transfection. Results of cell cycle analysis show that, Oct4/Sox2 adipose tissue derived MSCs in G1 were reduced with a concomitant rise in the fraction of cells in S phase. This indicates an acceleration in the transition of cells from G1 to S phase accompanied with higher differentiation abilities of the MSCs [68]. In an in vitro study, poly-l-lysine (PLL) was found to effectively prevent senescence and augment growth of MSCs [70]. This was achieved through the upregulation of genes involved in cell cycle, adhesion, stemness, proliferation, differentiation, and FGF-2 signaling. It is also reported that, the disruption of mitochondrial reactive oxygen species (mtROS) homeostasis is principal in inducing MSC senescence. Therefore, the prevention of mtROS accumulation aids in suppressing senescence [71]. On this background, EphB2 overexpression has been applied during which the EphB2 signaling increased MnSOD and reduced the mtROS level in MSCs, consequently optimizing MSCs therapeutic influence in wound repair [71].

Again, telomerase reverse transcriptase (TERT) transfected MSCs were reported to hinder senescence and possess higher proliferative and cell cycle-related gene expression factors [72], and as well enhanced neural and osteogenic lineages proliferation [73, 74]. Some of the interactions known to underlie the mechanism of the human TERT gene enhancing the self-renewal ability of MSCs (and averting cellular senescence) include the complex formation with molecules such as securin, heat shock protein 90 and chaperones such as Ku70 [72]. Signaling pathways involved in the modulatory functions of TERT gene to enhance osteoblast differentiation of human bone marrow-derived MSCs include insulin-like growth factor (IGF) signaling. Particularly, IGF-induced AKT phosphorylation and alkaline phosphatase (ALP) activity are known to aid osteoblast differentiation [74].

## Preconditioning modification

Preconditioning of MSCs encompasses the ex vivo treatment with both chemical and physical factors via specifically designed environment. Just as discussed above, preconditioning is also meant to maintain and enhance the intrinsic therapeutic properties of MSCs (Fig. 1) against the odds of hostility within its transplanted microenvironment. Furthermore, preconditioning is known to improve the interaction between MSCs and in innate/adaptive immune system. For example, hypoxia treated MSCs express more antiapoptotic proteins, IL-8 and IL-6 [75], as well as IL-10 and FasL [76]. The enhanced immunoregulation in turn dampens inflammation, and encourage regeneration and tissue repair.

### Improving migration

MSCs generally thrive in a low oxygen tension environment usually between 1–5% [77] or 1–7% [78] in vivo. However, in vitro cultivation strategies typically provide an average oxygen tension environment between 20 and 21% which may negatively impact their cellular functions. Hence one classical method of maintaining and/or enhancing MSC functions is via hypoxia preconditioning, a proven approach to augment migratory, proliferative, prosurvival genes and trophic factors expression properties of these cells [77, 79, 80]. The migratory property of MSCs are promoted using hypoxic preconditioning coupled with microbubble-mediated ultrasound. In this study, the expression levels of SDF-1 and CXCR4 increased in 24 h after conditioning, further upregulating SDF-1/CXCR4 expression and improving the migration ability of the MSCs [81]. Hypoxic preconditioning of MSCs, again evokes increase expressions of LincRNA-p21, alongside CXCR4/7 and HIF-1α, together promoting MSCs migratory capacity and survival [82].

On the background of their previous report that, IL-3 prevents bone and cartilage damage, and increases the differentiation potential of MSCs into functional osteoblasts, Barhanpurkar-Naik et al. [83] further investigate the role of IL-3 in the migration of MSCs. They report that MSCs conditioned with IL-3, overexpress CXCR4 which causes increased migration towards SDF-1α (i.e. SDF-1/CXCR4 axis). Using C57/B6 mouse model of liver ischaemia/reperfusion injury, the in vivo migration of rapamycin-preconditioned umbilical cord derived-MSCs have been studied. It was noticed that, the induction of autophagy by rapamycin promotes the capability of the MSCs to migrate and express anti-inflammatory cytokines as well as increase the expressions of CXCR4 without affecting cellular viability. Upon in vivo administration of the modified MSCs, more of the cells migrated towards the ischaemic regions through the CXCR4/CXCL12 axis resulting in improved hepatic function and reduced inflammatory cytokines [84]. Again, preconditioning MSCs with deferoxamine, is an efficient way of increasing migration as well as homing [85]. Oncostatin M (OSM) preconditioned MSCs have also been demonstrated to overexpress gp130/OSMRβ (type 2 OSM receptor) causing upregulation of HGF. The resultant increased cellular migration and proliferation ameliorates lung fibrosis in mice in 18 days post-transplantation [86].

### Improving adhesion

Just as elaborated earlier, hypoxic preconditioning of MSCs boosts their capacity to engraft and survive in target tissues. Liu and colleagues demonstrate that, MSCs express high levels of both SDF-1 receptors, CXCR4 and CXCR7 under 3% O_2_ concentration. These factors alongside the evoked expression of HIF-1α and phosphorylation of Akt, cause higher MSCs adhesion, migration and survival [87]. Bone marrow-derived MSCs were preconditioned with 2,4-dinitrophenol (DNP) in a rat model of myocardial infarction. The DNP-MSCs were found to express higher adhesion to the surface and increased viability resulting in significant cardiac function recovery. The researchers concluded that, the enhanced cardiac function post transplantation was due to heightened adhesion, survival, homing, as well as cardiomyogenic and angiogenic differentiation of the preconditioned MSCs [88]. Plasminogen activator inhibitor 1 (PAI-1) is known to negatively regulate MSCs survival in vivo. Mechanistically, PAI-1 extracted from MSCs does not influence MSCs survival via a plasmin dependent manner but rather direct impact on the adhesiveness of MSCs to their surrounding matrices. Preconditioning modifications aimed at inhibiting or knocking down PAI-1 produces enhanced MSCs adhesion and autograft survival [89].

### Improving survival

Under oxidative stress, MSCs are highly prone to apoptosis accompanied by reduced functional activity. In investigating factors that avert this effect, MSCs preconditioned with tumor necrosis factor-α (TNF-α) were found to not only promote MSCs survival, but also migration and proliferation to repair endothelium in intimal hyperplasia of vein grafts [90]. The signaling pathway implicated in this study is the NF-κB pathway. Lee et al. [78] also uncovered the mechanism involving 78-kDa glucose-regulated protein (GRP78) to elucidate the improved MSCs bioactivity and survival in hindlimb ischemia model. The expression of GRP78 under hypoxic (2% O_2_) preconditioning is greatly upregulated via the HIF-1α-GRP78-Akt signal axis, leading to enhanced survival, proliferation, migration and angiogenic cytokine secretion potential of transplanted MSCs. Preconditioning is known to even restore impaired functions of MSCs as demonstrated by Khan and colleagues in a diabetic affected MSCs using the growth factors IGF-1 and FGF-2 [91]. A similar report also indicates that, myogenic medium preconditioning improved MSCs survival, proliferation, angiogenic capability, alongside increased AKT phosphorylation. Four weeks post transplantation of the modified MSCs results in augmented functionalities such as decreased apoptosis and fibrosis, as well as increased angiogenesis within diabetic hearts [92].

On the basis of enhancing communication with the immune system to protect MSCs, treatment with interferon gamma (IFN-γ) produces increased suppression of NK cells activation, hence protecting MSCs from NK cells-mediated cytotoxicity [93], and the addition of TNF-α further improves MSCs function to express factor H, a key factor linked with the inhibition of complement system activation [94]. The suppression of NK activation is through prostaglandin-E2 secretion in a contact-independent manner, and IFN-γ-stimulated MSCs are less susceptible to NK killing [93]. It is also documented that hypoxic preconditioning at oxygen tensions of 1%, 2%, 3% and 4% show greater MSCs cellular complexity and decreased tendency to autophagy, enhancing their survival [95].

### Reducing premature senescence

Hypoxic preconditioned-MSCs exert superior protected role and has been confirmed to involve HIF1α and Beclin1 signaling pathway in several conditions [96–98]. Pretreatment of MSCs with different hypoxic oxygen concentrations have shown promising outcomes in the therapeutics of MCSs by critically influencing their inherent characteristics including reduced senescence. For instance, 1% O_2_ enhances proliferation, viability, stemness and chemokine related genes expression including CXCR7 and OCT4. It again prevents phenotypic changes in MSCs such as cellular morphology and the expression of senescence-associated-β-gal [80]. Furthermore, 2% O_2_ enhances bioactivity, proliferation and survival via the suppression of the cell death signaling pathway and augmentation of angiogenic cytokine secretion [78], while 5% O_2_ exhibits higher chondrogenic differentiation ability by preventing cellular senescence and promoting the proliferative capacity of human synovial MSCs [99].

Herbal extracts have also been used to precondition MSCs to examine their effects on senescence and proliferation properties of MSCs. In one of such studies, *Withania somnifera* root and *Tinospora cordifolia* leaf extracts were employed to modify Wharton’s jelly MSCs. Analysis shows delayed senescence, decreased apoptotic cells, increased proliferation and increased G2/M phase of the cell cycle [100]. The effects of organic nitrates like isosorbide dinitrate (ISDN) on senescent MSCs (induced by high glucose) have also been documented. It is reported that, ISDN preconditioning of senescent MSCs greatly decreases several senescence-related biomarkers, and as well reverses the downregulation of ERK activity and forkhead box M1 (FOXM1) expression in the MSCs. They further conclude that, the protective influence of ISDN against senescence of MSCs is via the activation of the ERK/FOXM1 signaling pathway and the upregulation of miR-130b [101]. Other molecules utilized in enhancing the therapeutics of MSCs in preconditioning modification include but not limited to Apelin 13 [102], IL-1β, TLR ligands, and lipopolysaccharide (LPS) [17]. Apelin, the endogenous ligand for the previously orphaned G protein-coupled receptor APJ is demonstrated to exert anti apoptotic effect on oxidative stress-induced apoptosis in MSCs through the MAPK/ERK1/2 and PI3K/AKT signaling pathways. This implicates that, pretreatment of hypoxic preconditioned MSCs with apelin 13 would be an efficient approach to modify and probably enhance MSCs efficacy [102].

## Therapeutic application of modified mesenchymal stem cells

**Fig. 2:**
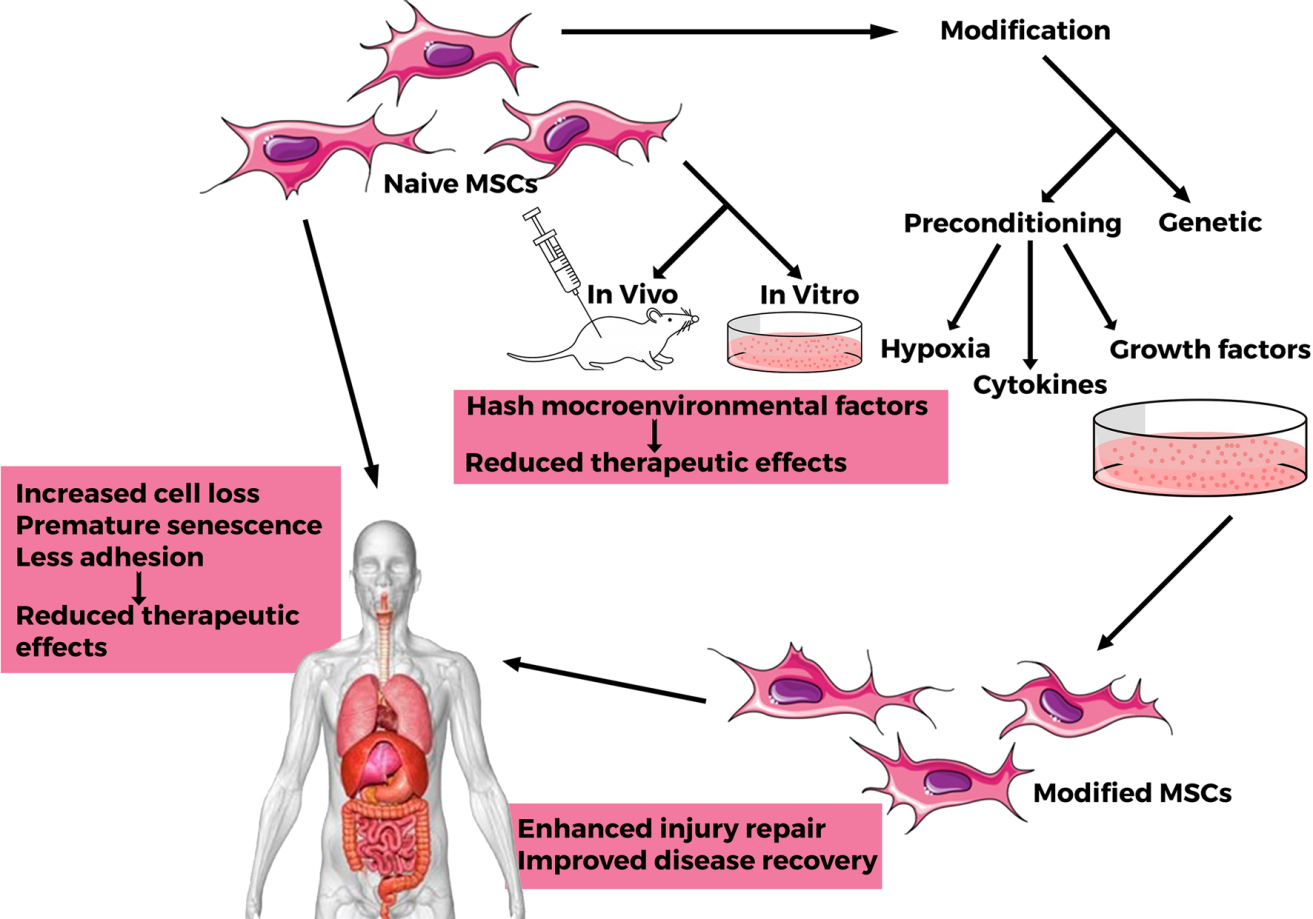
The cycle of naïve MSCs to modified MSCs towards clinical application. The ordinary MSC is confronted with several inhospitable factors that cause it to have reduced therapeutic effect. Upon preconditioning and/or genetic modification, they gain improved therapeutic functionalities of increased injury repair and disease recovery

With focus on circumventing in vitro and in vivo factors that cause the ordinary MSC to lose its intrinsic properties of proliferation, differentiation and survival, MSCs have been modified and applied in several diseases. The cycle of naïve MSCs to modified MSCs towards clinical application is illustrated in Fig. 2. In an instance of a hemophilia study, bone marrow derived MSCs transfected with human coagulation factor IX (hFIX) highly expressed hFIX with increased coagulation activity by 2.4- to 4.4-folds in contrast to other modified cells [103]. Genetic modification using Sox11, also results in enhanced migration of MSCs to a fractured bone site, speeding the healing process [104] while integrin α4 overexpression promoted transmigration, with a resultant reduced cerebral embolism [105]. Sox11 overexpression does not only increase the migration of MSCs but also the cell viability as well as their trilineage differentiation. Additionally, Sox11 activates the bone morphogenetic protein (BMP)/Smad signaling pathway, runt-related transcription factor 2 (Runx2) and CXCR4 expressions in MSCs. These activated factors together improve the Sox11-modified MSCs therapeutic effects. Furthermore, miR‐122 modification is noted to enhance the therapeutic efficacy of MSCs via exosome mediated miR‐122 communication in liver fibrosis [106]. Among the several other conditions in which modified MSCs have shown improved therapeutic outcome are Huntington’s disease [24], spinal cord injury [107], endothelial injury [108], as well as neurological, cardiovascular, respiratory and diabetic associated conditions as expanded below.

### Neurological conditions

MSCs are known to enhance neurogenesis and effect neuroprotection. During MSC transplantation in brain injury, it is expected that sufficient cells move into the injured site to exert enough therapeutic effect. This has made local application a common practice. However, direct local inoculations are confronted with brain tissue damage and induced inflammation [109], whereas cerebrospinal fluid spaces administration are also faced with scarce intra-parenchymal migration [110], hence inadequate therapeutic effect. Modification of MSCs in this field is meant to circumvent these challenges among others to ensure cyto-protection and neurogenesis [111]. MSCs modified to overexpress certain factors like brain derived neurotrophic factor (BDNF), greatly enhance motor functions and reduce brain lesion volume when administered intranasally. In this same study, the enhanced motor function was also seen in epidermal growth factor-like 7 (EGFL7) modified MSCs [112]. The observed outcome is due to regulation of differentiation and proliferation of neural stem cells. Huang and colleagues assessed the transplantation effects of umbilical cord derived MSCs overexpressing the chemotactic factors CXCR4 in lesion cavity of a rat brain. With an additional scaffold of human BDNF (brain-derived neurotrophic factor) linked to chitosan scaffolds, the modified MSCs adequately migrate to the target site, and induce tissue regeneration within the traumatic brain injury environment [113]. Again, SOD3-MSCs administration do not only reduce the infarct volume of ischemic stroke rats, but also significantly improve the neurological function [114]. The effects of exosomes extracted from hypoxic preconditioned MSCs on memory deficits in Alzheimer disease mice model have also been documented. Aside the effective regulation of cytokines within the environment, the hypoxic preconditioned MSCs exosomes effectively upregulate the levels of miR-21 in the brain of Alzheimer disease mice. This was accompanied with restored cognitive deficits in the APP/PS1 mice and inhibited pathologic features [115]. Additionally, the application of bone marrow-derived MSCs modified with BDNF gene (via adenoviral transduction), to ameliorate neurological deficits in ischemic stroke model of rats have also been investigated. Transplantation of these MSCs improve proliferation of endogenous neural stem cells while suppressing cell death. Further analysis reveals increased doublecortin (DCX-) positive neuroblasts and Neuronal Nuclei (NeuN-) positive mature neurons in the subventricular zone and ischemic boundary, resulting in greater neurological functional recovery in the rats [116].

### Cardiovascular diseases

The therapeutic utility of MSCs in cardiac conditions is based on the MSCs capability of directly differentiating into cardiac tissue, and as well release paracrine factors to promote infarct repair, regenerate damages, improve vascular supply and restore myocardium function. The use of MSC or cell therapy in general has been a promising approach in this direction. However, the overall restorative ability of these cells appear confined basically due to low engraftment, in addition to poor cell viability within the ischemic myocardium microenvironment. Huang and colleagues earlier hypothesized that, inadequate chemokine receptors on MSCs could be a factor for the deficiency in survival and engraftment [21]. In their experiment, genetically modified CCR1-MSCs dramatically augment MSCs migration, survival, and engraftment in the infarcted myocardium with superior therapeutics outcome compared with the controls. Intramyocardial administration of the modified MSCs causes decreased cardiomyocytes apoptosis, reduced infarct size, and also prevents cardiac remodeling with restored cardiac functions 4 weeks post induced-myocardial infarction [21]. In another study, increased MSCs survival, proliferation, angiogenesis and differentiation mediated by ILK-overexpressed MSCs, result in a general response of decreased fibrosis, scar size and apoptosis, and increased myocardial cell proliferation and perfusion [117]. The researchers note that, intracoronary administration substantially improves the homing ability of ILK-MSCs to infarct myocardium in the porcine. The cardio-protective effects of MSCs conditioned to overexpress HGF, is also evaluated in a mouse model of myocardial infarction. With higher expression levels of HGF, VEGF, EGF and bFGF, the modified MSCs were found to significantly protect cardiomyocytes and enhance overall cardiac function via less cardiomyocyte apoptosis, increased proliferation, as well as enhanced angiogenesis [54].

### Lung injury

Damage to any compartment of the lung due to chemical, physical, or biological factors/agents which ultimately leads to inflammatory reactions, constitute lungs injury. The induced injury affects cellular components such as DNA, proteins and lipids leading to cell death. Unfortunately, available medical management largely remain supportive, hence the need for countermeasures of which MSC therapy is promising. Among the several MSC therapy studies conducted in this field include a radiation induced lungs injury experiment, where Liu and colleagues noticed that, although the ordinary MSC decreases apoptosis and infiltration of lymphocyte, and as well increases epithelial cells proliferation and fibrosis inhibition in the later phase, the decorin-modified MSC produces a more enhanced therapeutic effect [118]. Similarly, manganese superoxide dismutase (MnSOD) gene modified MSCs significantly improve radiation induced lung damage, reduce inflammation and protect the cells from apoptosis [119]. In these experiments, the major markers analyzed include inflammatory cytokines levels in plasma, lung histopathology and Treg levels in the peripheral blood and spleen. The MnSOD-modified MSCs could differentiate into epithelial-like cells post transplantation in the mice, while the decorin-modified MSCs effectively induce interferon-γ expression, decreased Tregs levels and inhibited collagen type III α1 expression within pulmonary tissues [118, 119]. In other respiratory conditions, genetically modified MSC overexpressing angiotensin II type 2 receptor induce higher MSCs migration and accumulation at injured site, accompanied by substantial decrease in pulmonary vascular permeability as well as restored lung histopathology compared to the control group [120]. The treatment of a consolidated lung fibrosis with miRNAs let-7d modified MSCs elicits changes in cytokine expressions that alter the injury, cause weight gain and improve survival rate. Intravenous administration of the antifibrotic miRNAs let-7d-modified MSCs in the murine bleomycin model, also caused distinctive expressions of CD45-positive cells and slight decrease in collagen mRNA levels in the lungs [121]. Furthermore, hypoxia induced MSC therapy produces encouraging results in different respiratory injuries including pulmonary fibrosis [86, 122] and radiation induced injury [123]. Based on the background that hypoxia induces the expressions of cytoprotective genes and also encourages the secretion of anti-inflammatory, anti-apoptotic and anti-fibrotic factors, hypoxic preconditioning has been employed to increase survival time of engrafted MSCs. For example, intratracheal instillation of hypoxic modified MSCs (overexpressing HGF) are noted to significantly ameliorate lung fibrosis by attenuating extracellular matrix production via paracrine effects; downregulating fibrotic and inflammatory factors, and improving pulmonary respiratory functions [122].

### Diabetes and its associated conditions

Diabetes and its associated obesity are swelling in numbers at an alarming rate worldwide, resulting in increased co-morbidities. Although optimal management remains elusive, MSC therapy in this field has attracted a lot of attention with highly encouraging results. In obese diabetic mice model, intra-peritoneal administration of MSCs modified to overexpress enhanced green fluorescent protein (eGFP) and superoxide dismutase (SOD2), effectively home in inflamed fatty pockets, consequently improving glucose tolerance and reducing total body weight at week 4 [124]. Similarly, respective cytosolic and mitochondrial antioxidant genes catalase and Sod2 modified MSCs given intraperitoneally in mice, improve diabetes associated fatty liver disease and decrease systemic inflammation with enhanced glucose tolerance [125]. These antioxidant preconditioned-MSCs elicit these effects by reducing oxidative stress, adipose tissue inflammation, white fat hyperplasia and mitochondrial dysfunction, which are known to be linked with obesity associated with type 2 diabetes. Intravitreal inoculation of pigment epithelial-derived factor (PEDF)-MSC exerts protective effect on nerves in diabetic retinopathy [126] while pretreatment of hypoxia preconditioned MSC with apelin 13 improves their therapeutic efficacy in a diabetic model as earlier expanded [102]. Again, MSC-based nanocarrier therapy repairs a diabetic wound by increasing cellular adhesion and proliferation of MSCs, as well as their differentiation into osteoblasts and adipocytes, producing a resultant collagen deposition and angiogenesis. This new collagen-nanomaterial-drug hybrid scaffold provides new prospects for application in efficient stem cells based therapy, therapeutic drug delivery, tissue engineering and regenerative medicine [127]. MSC are also known to repair chronic injuries caused by hyperglycemia by protecting β cells via regulation of autophagy [128] and express biosensor proteins for monitoring blood glucose concentration [129]. Siska et al. [129] genetically modified MSCs co-expressing hTERT and a secreted glucose biosensor transgene by utilizing the Sleeping Beauty transposon technology. Increased activities of hTERT is also complemented by constant level of stem cell pluripotency marker. MSCs modified to optimize their anti-inflammatory effects are also applied in the management of painful diabetic peripheral neuropathy. The treated mice were observed to have improved behavior and reduced serum levels of several pro-inflammatory cytokines, resulting in significantly attenuated symptoms of painful diabetic peripheral neuropathy [130]. Summary of modified MSCs application in some selected conditions are presented in Table 1.

**Table 1:** Selected cases of modified MSCs application and therapeutic outcome

Modification element	Condition	Results	References
Decorin	Radiation-induced lung injuries	↓Lymphocyte infiltration and apoptosis ↑Proliferation of epithelial cells and inhibition of fibrosis Effective induction of interferon-γ expression and inhibition of collagen type III α1 expression	[118]
miRNA	Lung fibrosis	Distinctive expression in cytokines and CD45-positive cells Quick weight gain and improved survival rate	[121]
CCR1	Injured myocardium	Augmented cell survival, migration and engraftment Significant reduction in infarct size, reduced cardiomyocytes apoptosis and increased capillary density in injured myocardium Inhibited cardiac remodeling and restored cardiac function 4 weeks after injury	[21]
Betacellulin	Hyperglycemia	Increased insulin production levels Ameliorated hyperglycemia	[131]
Deferoxamine	Diabetes	Upregulation of CXCR4/CCR2 and higher activity of MMP-2/MMP-9 Increase migration and subsequent homing	[85]
Sox11	Bone fracture	Increased differentiation, migration, and viability of MSCs under oxidative stress	[104]
		Activate of BMP/Smad signaling pathway, Runx2, and CXCR4 Accelerating bone fracture healing	
ACE2	Injured endothelium	Decreased inflammatory mediators, reduced paracellular permeability, and enhanced survival of endothelial cells Restore endothelial function	[108]
Integrin α4	Cerebral embolism (stroke)	Increased transendothelial migration of MSCs Decreased cell aggregation and ameliorated evoked cerebral embolism in stroke rats	[105]
miR‐122	Liver fibrosis	Suppressed the proliferation of and collagen maturation in HSCs Enhanced the therapeutic efficacy of MSCs	[106]

Different molecules and factors have been associated with modifying MSCs towards encouraging better therapeutic outcome. Applied modified MSCs have been documented across many human conditions in the experimental settings with promising results. This table enlists some of these diseases, elements used in the modification, and the key resultant outputs upon modified MSCs administration

*CCR1* c-c chemokine receptor type 1, *Sox11* sry-related high-mobility group box 11, *BMP* bone morphogenetic protein, *Runx2* runt-related transcription factor 2, *CXCR* cxc chemokine receptor, *ACE2* angiotensin‐converting enzyme 2, *HSCs* hepatic stellate cells, *MMP* matrix metalloproteinase

## Discussion and future perspectives

Under normal circumstances, ordinary MSCs may lose their intrinsic functions due to long period of in vitro cultivation, poor isolation procedure, and harsh environmental, and death signals upon in vivo inoculation. Modification of these cells genetically or by preconditioning does not only cause the retention of their characteristic properties, but also enhance their overall efficacy in therapeutics. One major short coming of MSC transplantation is their inability to sufficiently migrate and get engrafted or concentrate at the injured site, sometimes due to entrapment within lung and lymphoid organs. This has contributed to local inoculation currently being the most preferred method of administration [121]. However, this method has a lot of setbacks since its application in fragile organs like the brain, results in damage to tissue and increased pressure on indigenous structures causing inflammation and micro-bleeding [109]. This challenge is partly overcome via the application of modified MSCs which does not just improve migration and homing at target organs, but also increase cellular replacement repair as well as the overall paracrine and endocrine effects of transplanted MSCs.

Practically, MSC therapy occurs in a damaged cellular setting, where the microenvironment is harsh and embodies several destructive factors. With focus on the need to ensure that transplanted cells capably cope within such an environment by surviving enough to impact sufficient therapeutic influence, MSCs have been modified [132]. A lot of such studies focused on the modifications that help MSCs avert premature senescence and maintain their inherent therapeutic properties against inhospitable microenvironment. Again, optimal adhesion of MSCs which is crucial in the determination of their proliferation and viability within the transplanted environment has been the aim of several other researches. Altogether, the improvement of these focal characteristics affect and largely interdepend on each other (Fig. 1).

The unmodified MSCs as a therapeutic tool is again confronted with the challenge of lack of standardization leading to therapeutic discrepancies. A review paper by Galipeau, elaborated the lack of standardized MSC products throughout academic centers and industries to include donor variance, senescence and epigenetic reprogramming followed by culture expansions, induced immunogenicity during culture and cryopreservation [133]. Notwithstanding, the application of the modified MSC is also confronted with similar challenges and setbacks. While seeking to address these challenges, the issue of quality control measures on isolating, culturing and modifying MSCs should also be well defined and strengthened. Additionally, the route of administration, quantity of modified cells to be given, and the frequency of transplantation are worth manipulating in define studies since they capably influence MSC therapeutic outcome. In the future, optimization of culture media needs further exploration to ensure more stable modified MSCs void of accumulated genetic and epigenetic changes. Although the genetic information transfected into the host are generally stably integrated, there is still the need to carefully monitor random genomic integration in hosts. Random integration could cause critical genomic dysfunction and possible mutations leading to malignancies. Therefore the safety and long term effects of transplanted modified MSCs must be emphasized. Furthermore, mechanisms underlying the phenomenon of gene silencing should be well investigated and understood to avert the occasions of genetically modified MSCs turning off in a short time after transplantation.

Irrespective of the successes of modified MSCs therapy, excessive loss of transplanted cells still remain an unresolved challenge. This is the result of both in vitro and in vivo detrimental environmental factors or lack of favorable factors, inducing premature senescence and loss of survival signals. This calls for mounting strategies that targets specific molecular mechanisms or pathways and by inducing a broader scope of cytoprotection. Again, combined modification techniques and molecules/factors that enhance MSC survival, migration, homing, and adhesion could further optimize cell survival and maximize therapeutic effects. Finally, specific secretomes expressed by modified MSCs are largely responsible for the transplanted effects. Further identification, characterization and subsequent application of such therapeutic factors could yield more effective clinical outcomes.

## Conclusion

The importance of prosurvival and promigratory abilities of MSCs in their therapeutic applications can never be overemphasized. Again, their capability to adequately home at injury site and effectively differentiate, proliferate and enact their paracrine and endocrine influences, are the desired outcome of MSC based therapy. These effects are maintained and even further improved via MSC modifications. By and large, MSC modifications have produced better therapeutic impact with high specificity to targets than the ordinary cell. However, more studies on the combination of different genetic modification and/or preconditioning techniques or factors, leading to a combined application of these modified MSCs would ensure better outcome and novel discoveries, and further enhanced therapeutic goals. Again, there is the need to mount more focal investigations to identifying and understanding the many factors that influence MSC modification.

## Data Availability

Not applicable.

## Abbreviations

MSCs: Mesenchymal stem cells
CXCR: CXC chemokine receptors
CCR: C-C chemokine receptor
ITGA: Integrin subunit alpha
FAK: Focal adhesion kinase
VEGF: Vascular endothelial growth factor
ILK: Integrin-linked kinase
HIF: Hypoxia inducible factor
HGF: Hepatocyte growth factor
TNFR: Tumor necrosis factor receptor
TNF: Tumor necrosis factor
PLL: Poly-l-lysine
TERT: Telomerase reverse transcriptase
BFGF: Basic fibroblast growth factor
IL: Interleukins
FasL: Fas ligand
IGF: Insulin-like growth factor
FGF: Fibroblast growth factor
IFN: Interferon
hFIX: Human coagulation factor IX
BDNF: Brain derived neurotrophic factor
EGF: Epidermal growth factor
EGFL: Epidermal growth factor-like
DNA: Deoxyribonucleic acid
MnSOD: Manganese superoxide dismutase
eGFP: Enhanced green fluorescent protein
SOD: Superoxide dismutase
PEDF: Pigment epithelial-derived factor
Sox11: Sry-related high-mobility group box 11
BMP: Bone morphogenetic protein
Runx2: Runt-related transcription factor 2
ACE2: Angiotensin‐converting enzyme 2
HSCs: Hepatic stellate cells
MMP: Matrix metalloproteinase
CK1: Casein kinase 1
GSK3: Glycogen synthase kinase 3
mtROS: Mitochondrial reactive oxygen species
ILK: Integrin-linked kinase
DNP: 2,4-Dinitrophenol
OSM: Oncostatin M
gp130/OSMRβ: Glycoprotein 130/oncostatin M receptor beta complex
PAI-1: Plasminogen activator inhibitor 1
SDF-1: Stromal cell-derived factor-1
